# A retrospective observational study of intraductal breast papilloma and its coexisting lesions: A real‐world experience

**DOI:** 10.1002/cam4.3308

**Published:** 2020-08-21

**Authors:** Xiaona Li, Huan Wang, Zhe Sun, Chuifeng Fan, Feng Jin, Xiaoyun Mao

**Affiliations:** ^1^ Department of Breast Surgery The First Affiliated Hospital of China Medical University & School of Fundamental Science China Medical University Shenyang People’s Republic of China; ^2^ Department of Gynecology The First Affiliated Hospital of China Medical University Shenyang People’s Republic of China; ^3^ Department of Breast Surgery The First Affiliated Hospital of China Medical University Shenyang People’s Republic of China; ^4^ Department of Pathology The First Affiliated Hospital and College of Basic Medical Sciences of China Medical University Shenyang People’s Republic of China

**Keywords:** breast cancer, intraductal breast papilloma, real‐world experience

## Abstract

**Background:**

Breast intraductal papilloma is a heterogeneous group. The aim of the study is to investigate the intraductal breast papilloma and its coexisting lesions retrospectively in real‐world practice.

**Methods:**

We retrospectively identified 4450 intraductal breast papilloma and its coexisting lesions.

**Results:**

About 18.36% of intraductal papilloma coexisted with malignant lesions of the breast, 37.33% coexisted with atypia hyperplasia (AH), 25.24% coexisted with benign lesions, and only 19.10% coexisted without concomitant lesions. In addition, 36.80% of intraductal breast papilloma had nipple discharge, 51.46% had a palpable breast mass, and 16.45% had both nipple discharge and a palpable breast mass. About 28.18% experienced discomfort or were asymptomatic. Furthermore, 98.99% had ultrasound abnormalities, and 53.06% had intraductal hypoechogenicity upon ultrasound. 31.89% had mammographic distortion, and 14.45% had microcalcification upon mammography. Intraductal breast papilloma with malignancy had significant correlations with clinical manifestations.

**Conclusion:**

Coexisting malignancy was also related to ultrasound abnormality (BIRADS 4C and 5), mammographic distortion, and microcalcification upon mammography but was not related to the intraductal hypoechoic upon ultrasound. Coexisting atypical hyperplasia correlated with nipple discharge but not palpable mass, mammographic distortion, or intraductal hypoechoic upon ultrasound. The coexisting AH was also related to abnormality upon ultrasound or microcalcification compared with the benign lesions. The intraductal papilloma coexists with malignancy or AH accounted for more than 50%, and the clinical information on papilloma and its coexisting lesions is nonspecific. We recommended surgical treatment for benign intraductal papillary lesions.

## Graphical abstract

The current study provides insight into the real‐world pattern of the intraductal papillomas and their coexisting lesions in a population of Chinese women. We thought it is an important research on papilloma and breast cancer prevention.

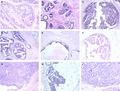

## 1 INTRODUCTION

An intraductal papilloma is a benign, or noncancerous breast tumor which originated from the epithelium of mammary ducts that forms in a milk duct. A fibrovascular stroma supported both the luminal epithelial and the outer myoepithelial cell layers for the formation of epithelial fronds, which is a characteristic of the intraductal papilloma.^1^ The morphologic changes of epithelial component include from metaplasia to hyperplasia, atypical intraductal hyperplasia, or in situ carcinoma.^2^ The symptoms, clinical signs, and supersonical appearances widely differ. These lesions may present clinically either as ultrasound abnormalities or palpable breast masses, with or without nipple discharge. Central papilloma originates from large ducts, often accompanied by pathological nipple discharge, while most peripheral papillomas occur in terminal ductal‐lobular unit (TDLU), involving small ducts.^3^, ^4^ The prognosis and treatment of papillomas have been influenced by views on their “precancerous” potential, and papillomas can harbor occult atypia hyperplasia (AH) or carcinomas. The management of benign intraductal papilloma remains controversial because of its nonspecific clinical findings, as well as its association with surrounding malignant pathology.^5^, ^6^, ^7^ Herein, we present the results from an observational study carried out according to a retrospective design. Our study aimed to address intraductal breast papilloma and its coexisting lesions.

### 1.1 Study design

In this study, patients with intraductal papilloma of the breast who underwent surgical resection in the First Affiliated Hospital of China Medical University from November 1999 to July 2017 were analyzed retrospectively. During the period of 18 years, a total of 5708 women were examined. Two pathologists, with a subspecialty focus on breast lesions, reviewed each case independently. Only those cases where both pathologists had the same diagnosis can be used. Of those, 4450 cases were eligible and selected into the research queue, according to the criteria of agreement and ethical approval. We reviewed the clinicopathological data of each patient. The patients before February 2010 did not sign informed consent due to the exemption of the retrospective format. All patients after February 2010 involved in the study signed informed consent to participate in the study and agreed to publish the results.

### 1.2 Patients

We retrospectively identified 4450 intraductal breast papilloma patients with surgical excision from November 1999 through July 2017 at the First Affiliated Hospital of China Medical University. The patients’ ages ranged from 13 to 88, with an average age of 47.86 ± 11.93 years.

### 1.3 The concomitant lesion of the breast

We classified all pathological types of breast lesions according to the World Health Organization standards published by Tavassoli FA et al.^8^, ^9^ A comprehensive list of pathologic features was reviewed, including margins whose tissue‐free ranges were defined as < 15 mm; concomitant adenosis; AH (including atypia ductal hyperplasia and atypia lobular hyperplasia); ductal or lobular carcinoma in situ; and invasive ductal cancer and other malignancies.

### 1.4 Statistical analysis

All the statistical analyses were descriptive in nature. The independent two‐sample *t* test was performed to evaluate continuous categorical variables between benign, atypical, and malignant lesions. The Chi‐square or Fisher's exact tests were used for comparing categorical variables. One‐way ANOVA was used to identify the association between different groups and patients’ age. A *P* value < .05 was considered statistically significant.

### 1.5 Ethics

The research was reviewed and approved by the Ethics Committee of the First Affiliated Hospital of China Medical University. All of the methods were performed in accordance with the Declaration of Helsinki and the relevant guidelines.

## 2 RESULTS

**FIGURE 1:**
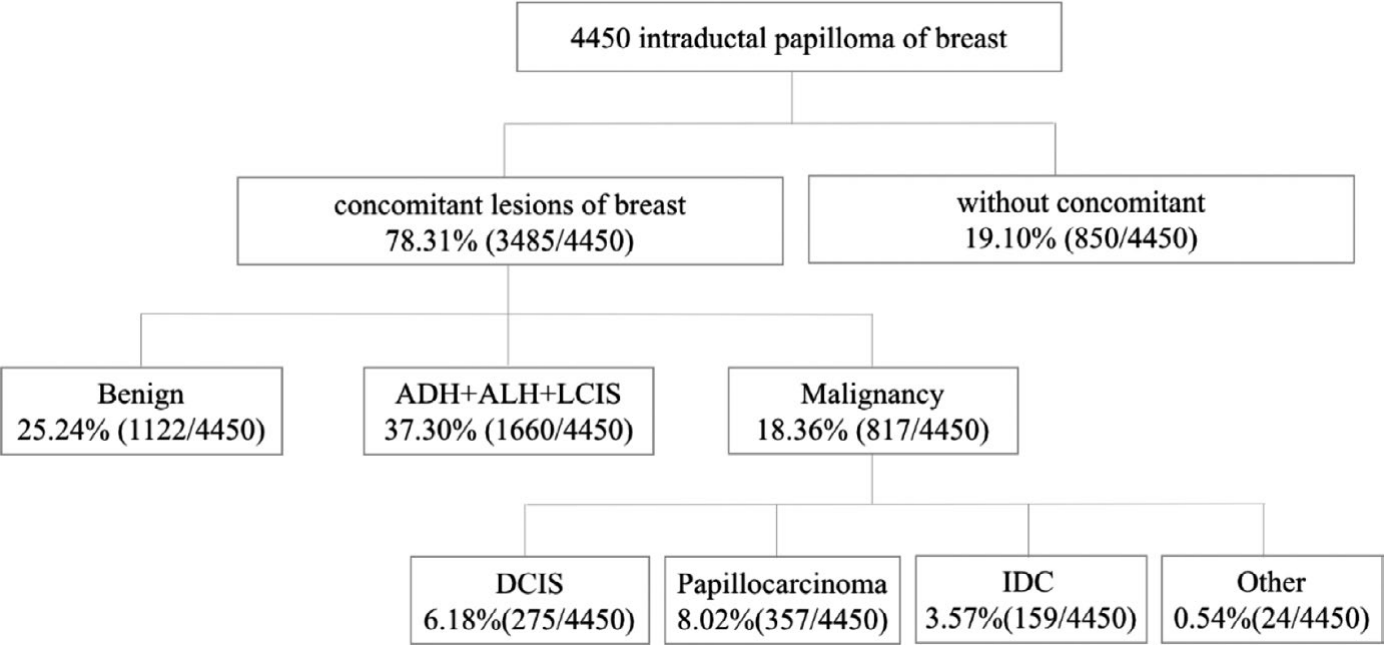
The papilloma and its coexisting breast lesions. This chart indicated that a total of 18.36% (817/4450) of intraductal breast papilloma coexisted with malignant lesions of the breast, 37.30% (1660/4450) coexisted with AH (including ADH and ALH), 25.24% (1123/4450) coexisted with benign lesions, and only 19.10% (850/4450) coexisted without concomitant lesions

**FIGURE 2:**
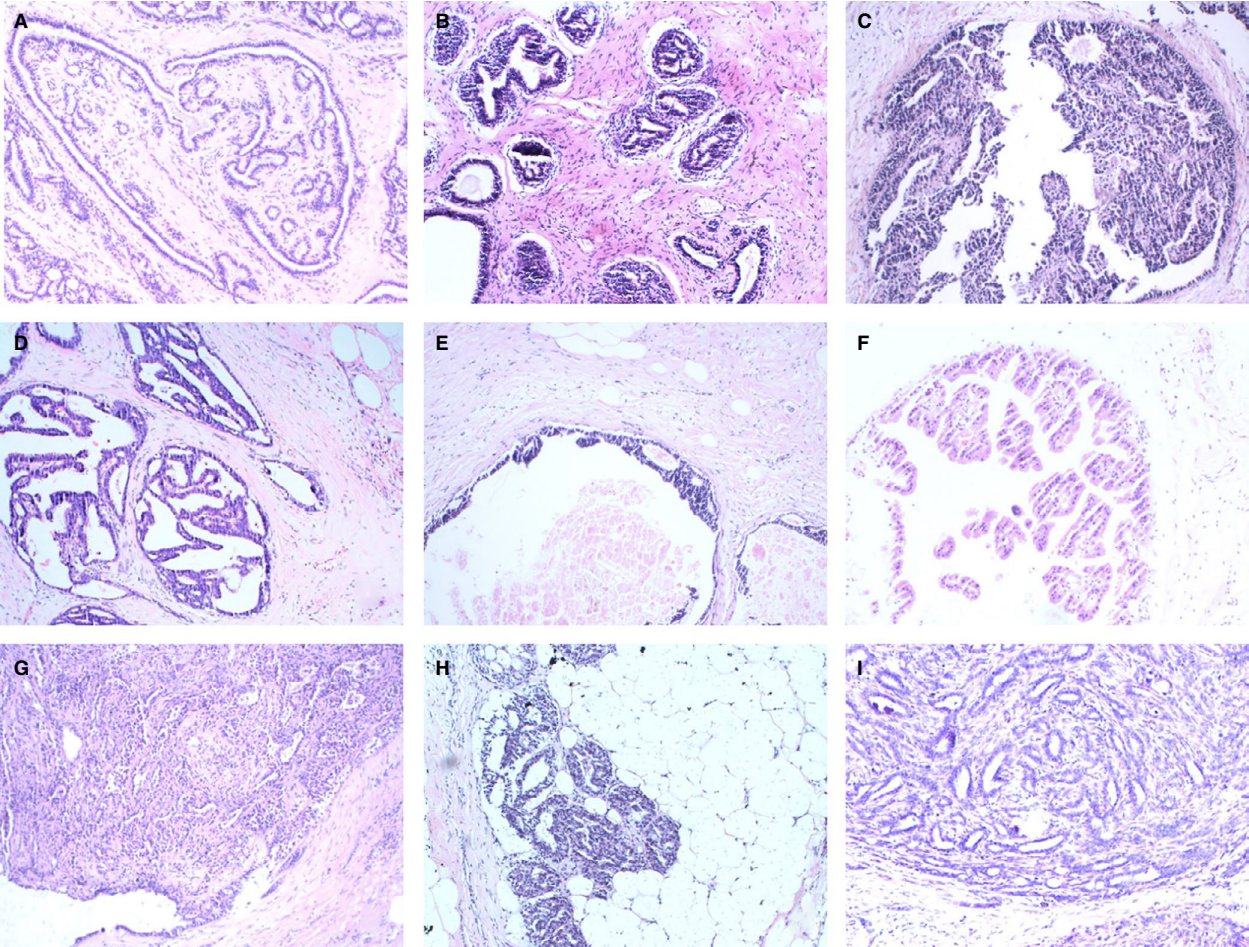
The papilloma and its coexisting benign lesions. A‐C, pure papilloma. D, papilloma and its coexisting usual ductal hyperplasia. E, papilloma and ductal ectasia. F, papilloma and apocrine metaplasia. F, papilloma and fibroadenoma. G, infarcted papilloma. H, papilloma and adenomyoepithelial adenosis. I, papilloma with simple epithelium and complex glands

**FIGURE 3:**
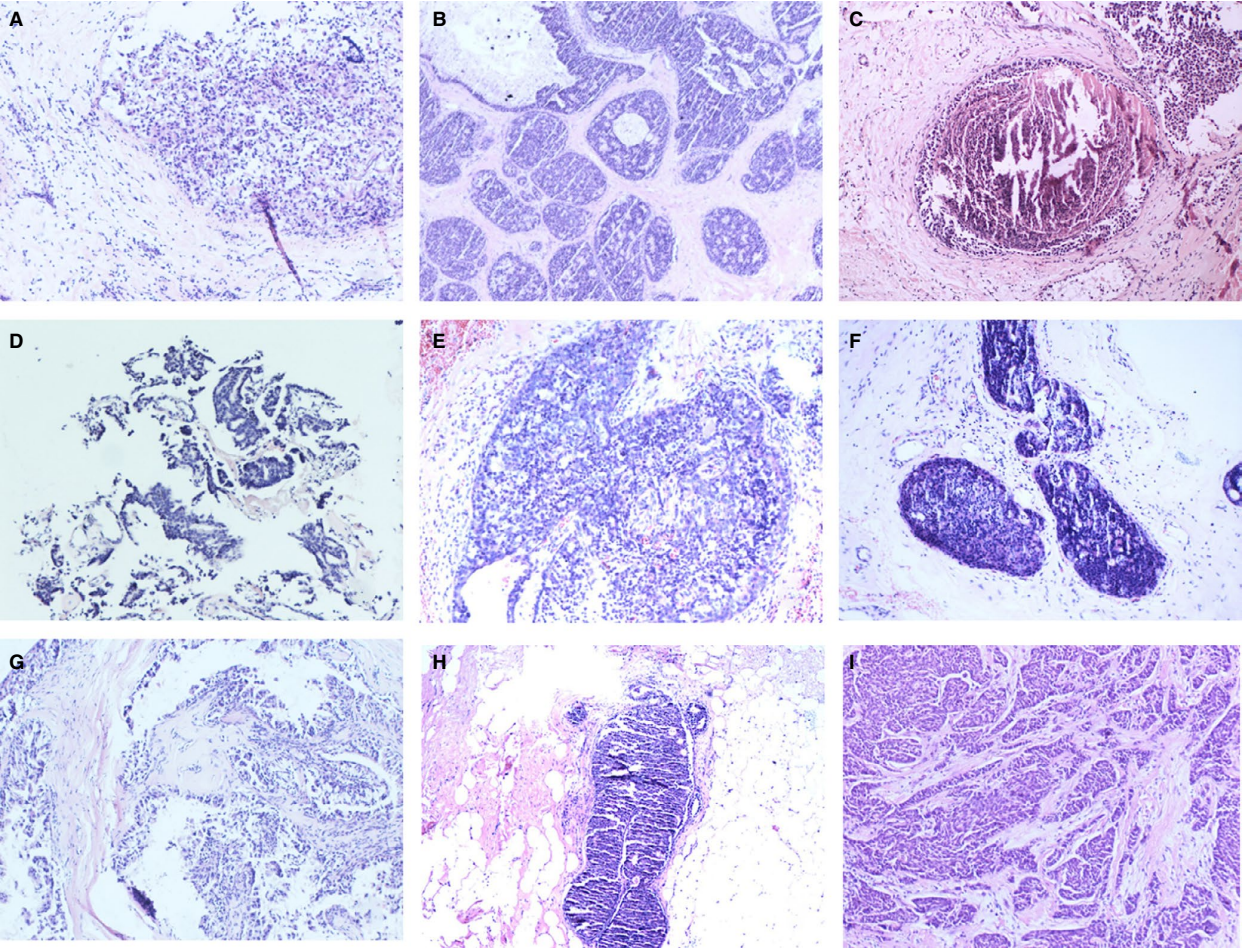
The papilloma and its coexisting ductal carcinoma in situ

**FIGURE 4:**
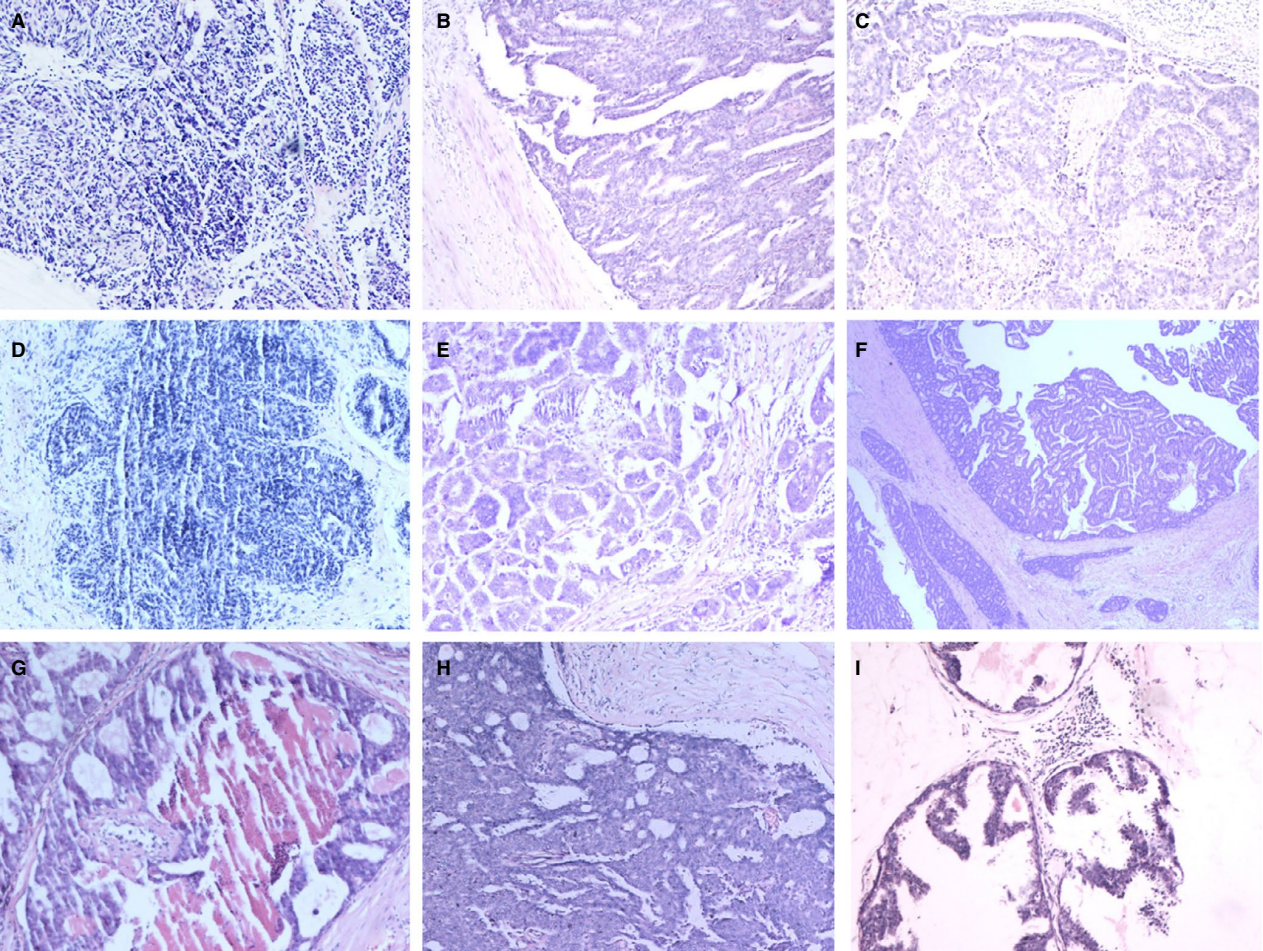
Papillocarcinoma

**FIGURE 5:**
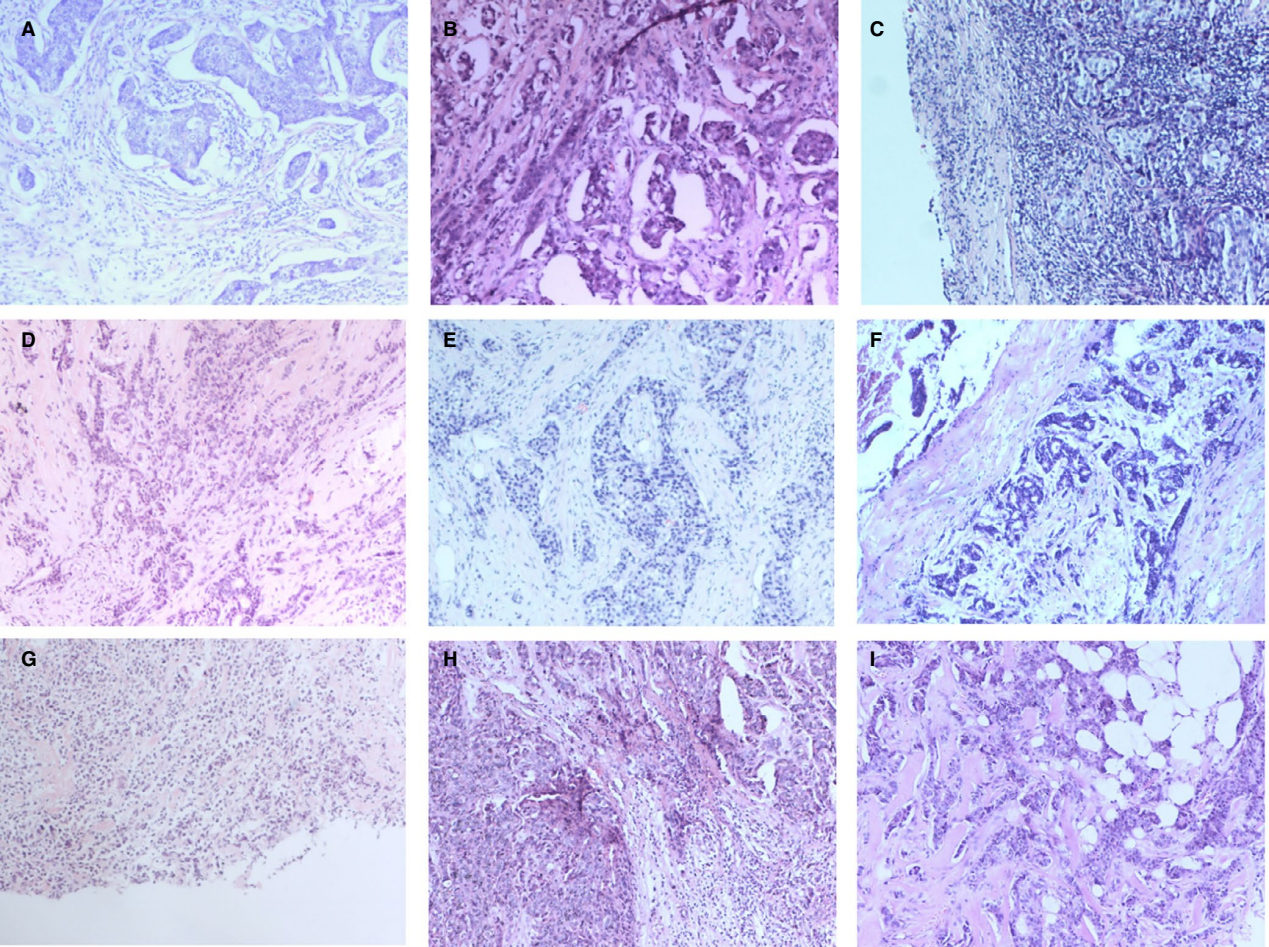
The papilloma and its coexisting invasive ductal carcinoma

A total of 18.36% (817/4450) of intraductal breast papilloma coexisted with malignant lesions of the breast, 37.30% (1660/4450) coexisted with AH (including atypia ductal hyperplasia [ADH] and atypia lobular hyperplasia [ALH]), 25.24% (1123/4450) coexisted with benign lesions, and only 19.10% (850/4450) coexisted without concomitant lesions, Figure 1. Figure 2 shows the papilloma and its coexisting benign lesions. Figure 3 shows the papilloma and its coexisting ductal carcinoma in situ (DCIS). Figure 4 shows the typical papillocarcinoma. Figure 5 shows the papilloma and its coexisting invasive ductal carcinoma. For the coexisting benign lesions, 157 cases coexisted with fibroma. The clinical symptoms of papilloma and its coexisting lesions including asymptomatic lesions, nipple discharge, and a palpable mass are nonspecific. A papilloma usually involves nipple discharge, which contains serous fluid sometimes or blood and both. In our results, 36.80% (1638/4450) of the intraductal breast papilloma had nipple discharge, and 51.46% (2290/4450) had a palpable breast mass. A total of 16.45% (732/4450) had both nipple discharge and a palpable breast mass. In addition, 28.18% (1254/4450) experienced discomfort in their breast or were asymptomatic, without nipple discharge or a palpable mass.

The radiological results of all the cases were recorded, and ultrasonography and mammography results included. A total of 11.51% (512 /4450) of patients did not have a mammography if they were ≤ 35 years old and had BIRADS 4A, confirmed by ultrasound abnormalities. In addition, 98.99% (4405/4450) had ultrasound abnormalities, and 53.06% (2361/4450) had intraductal hypoechogenicity by ultrasound. Furthermore, 31.89% (1256/3938) had mammographic distortion, and 14.45% (569/3938) had microcalcification upon mammograms.

In this cohort, the intraductal breast papilloma with malignancy was related to clinical manifestations, which contain a palpable breast mass (*P < *.01), concurrent nipple discharge, and a palpable mass (*P* < .01). Coexisting malignancy had significant correlations with ultrasound abnormality (BIRADS 4C and 5) (*P* < .01), but mammographic distortion and microcalcification upon mammograms had no correlations with intraductal hypoechogenicity by ultrasound (Table 1).

**TABLE 1:** The clinical characteristics of 4450 intraductal papilloma patients with surgical excision

characteristics	n	Pure papilloma or with nonmalignancy n = 3633	With malignancy n = 817	*P*‐value
Age, mean (SD)	4450	45.70 ± 11.19 (13‐88)	53.73 ± 12.63 (25‐86)	F = 280.65 *P* < .05
Nipple discharge				*χ* ^2^ = 0.29 *P* > .59
Yes	1638	1344	294	
No	2812	2289	523	
With palpable mass				*χ* ^2^ = 644.02 *P* < .01
Yes	2290	1542	748	
No	2160	2091	69	
Nipple discharge and mass concurrently				*χ* ^2^ = 158.68 *P* < .01
Yes	732	477	255	
No	3718	3156	562	
Ultrasonic abnormality				*χ* ^2^ = 28.27 *P* < .01
Yes	4405	3610	795	
No	45	23	22	
Intraductal hypoechogenicity by ultrasonic				*χ* ^2^ = 1.09 *P* > .05
Yes	2361	1941	420	
No	2089	1692	397	
Abnormality by ultrasonic n = 4405				*χ* ^2^ = 1537.89 *P* < .01
BIRADS 4A	3015	2872	143	
BIRADS 4B	911	619	292	
BIRADS 4C or 5	479	119	360	
Mammographic distortion n = 3938				*χ* ^2^ = 315.73 *P* < .01
Yes	1256	793	463	
No	2682	2348	334	
Microcalcification n = 3938				*χ* ^2^ = 1198.17 *P* < .01
Yes	569	147	422	
No	3369	2994	375	

In the subgroup of coexisting benign lesions and atypical lesions, coexisting atypical hyperplasia correlated with nipple discharge (*P* < .05) but did not correlate with a palpable mass (*P* > .05), mammographic distortion, or intraductal hypoechogenicity by ultrasound (*P* > .05). Coexisting AH was also correlated with abnormalities found by ultrasound or microcalcification (*P* < .01) compared with the benign lesions (Table 2).

**TABLE 2:** The clinical characteristics of 3633 intraductal papilloma patients with coexisting benign or atypical lesions with surgical excision

characteristics	N	Pure papilloma or with benign n = 1973	With AH or LCIS n = 1660	*P*‐value
Age, mean (SD)	3633	44.01 ± 10.50 (13‐81)	47.77 ± 11.50 (15‐88)	*F* = 92.61 *P* < .05
Nipple discharge				*χ* ^2^ = 6.48 .01 < *P*<.05
Yes	1344	693	651	
No	2289	1280	1009	
With palpable mass				*χ* ^2^ = 1.10 *P* > .05
Yes	1542	853	689	
No	2091	1121	971	
Nipple discharge and mass concurrently				*χ* ^2^ = 0.36 *P* > .05
Yes	477	253	224	
No	3156	1720	1436	
Intraductal hypoechogenicity by ultrasonic				*χ* ^2^ = 0.07 *P* > .05
Yes	1941	1058	883	
No	1692	915	777	
Abnormality by ultrasonic n = 4405				*χ* ^2^ = 1537.89 *P* < .01
BIRADS 4A	2872	1711	1161	
BIRADS 4B	619	199	420	
BIRADS 4C or 5	119	50	69	
Mammographic distortion n = 3141				*χ* ^2^ = 0.58 *P* > .05
Yes	793	415	378	
No	2348	1192	1156	
Microcalcification n = 3938				*χ* ^2^ = 21.14 *P* < .01
Yes	147	48	99	
No	2994	1559	1435	

**FIGURE 6:**
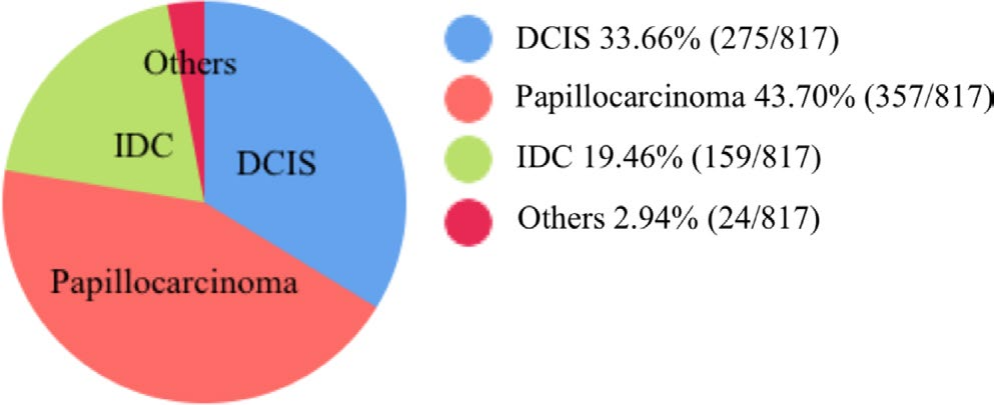
The coexisting malignancy of papilloma. This chart explained that 43.7% (357/817) were papillocarcinoma, 33.66% (275/817) were DCIS, 19.46% (159/817) were invasive ductal cancer of the breast, and 2.94% (24/817) were another type in the concomitant malignancy of intraductal breast papilloma

In the concomitant malignancy of intraductal breast papilloma, 43.7% (357/817) were papillocarcinoma, 33.66% (275/817) were DCIS, 19.46% (159/817) were invasive ductal cancer of the breast, and 2.94% (24/817) were another type. Figure 6 lists the types of coexisting malignancies.

## 3 DISCUSSION

Intraductal papillomas are benign tumors of the breast which arise from the epithelium of the lactiferous ducts (ie, a system that links the lobules of the mammary gland with the tip of the nipple).^10^ Intraductal papillomas require histopathological confirmation because it may turn out carcinogenesis.^11^, ^12^ Studies have also shown that intraductal papilloma with atypia increases the risk of DCIS or invasive breast cancer.^13^, ^14^ The treatment of breast intraductal papillomas has been controversial. Some advocate surgical resection of all lesions, despite benign pathological features, while others advocate removal only of specimens that are atypical or malignant. However, only a few studies have investigated this type of breast lesion and its coexisting cancers in Chinese women. We retrospectively reviewed 4450 intraductal papilloma of breast with surgical excision in Chinese women. The current study provides insight into the real‐world pattern of intraductal papillomas and their coexisting lesions in a population of Chinese women.

Core needle biopsy (CNB) plays an important role in the diagnosis of breast lesions. This biopsy is usually performed earlier than any other treatment. CNB has limitations, such as the incomplete removal of the representative sampling of the target.^15^, ^16^, ^17^ Published data were reviewed and provide estimates of the accuracy of percutaneous CNB and the upgrade to malignancy in diagnosing papillary breast lesions. Previous results strongly suggest that the diagnosis of intraductal papilloma by CNB should be surgical excision because a large number of lesions escalate into atypical lesions and malignant tumors at the time of resection.^18^, ^19^ The data in these studies vary widely, especially in cases of intraductal papilloma with malignant tumors. Tabulated data from the investigations done by Mercado CL et al (Table 3). The literature contained data demonstrating papillomas with benign findings in approximately 72% of cases, with atypical ductal or lobular cells (ADH, ALH, or lobular carcinoma in situ [LCIS]) in approximately 14%, and with DCIS or invasive carcinoma in approximately 13%, which were diagnosed by CNB and subsequent surgical resection. Wen X et al recently reported a meta‐analysis that contains 34 studies including 2236 nonmalignant breast papillary lesions at CNB that were histologically reviewed after surgical resection.^20^ In this research, 7.0%‐36.9% cases of benign papillomas were upgrade diagnosed as atypical papillary lesions and 15.7% cases of nonmalignant papillary lesions were upgrade diagnosed as malignancy. Three factors showed significant correlations with higher underestimation, including atypical papillary lesions (*P* < .001), positive mammographic results (*P* = .022), and published earlier than 2005 (*P* < .05). Our study showed that 18.36% of intraductal papillomas coexist with malignancy, and 37.33% coexist with ADH, ALH, or LCIS. The rate of coexistence with AH or LCIS is obviously higher than the rate previously indicated in Table 2. Most of the previous studies surveyed intraductal papilloma diagnosed with CNB and evaluated their potential coexisting risk of associated AH and malignancy, as identified by follow‐up surgical excision. Some research excluded part of the data of intraductal papilloma coexisting with malignancy or AH diagnosed with CNB. The patients in our research had a diagnosed papilloma by surgical excision without CNB. The data were the real‐world description data on intraductal papilloma and its coexisting lesions in China. If a patient had a diagnosed papilloma, she had an upgrade rate of 60% with ADH, ALH, or LCIS or with malignancy after undergoing surgical excision in China. Thus, we recommend surgical resection of benign intraductal papillary lesions with large range as a more aggressive treatment approach. If the range of lesions is small and limited, we also recommend the use of ultrasound‐guided excision to achieve both diagnosis and resection of breast intraductal papillary lesions.

**TABLE 3:** The coexisting breast lesions of intraductal papillomas: A selected literature review

Reference	Cases	Excision findings		
		Nonconcomitant or with benign n(%)	With atypical n (%)	With malignant n(%)
Mercado CL^19^	36	26 (72.22%)	8 (22.22%)	2 (5.56%)
Kil WH^45^	76	62 (81.58%)	5 (6.58%)	9 (11.84%)
Rizzo M^46^	169	109 (64.50%)	19 (11.24%)	41 (24.26%)
Bertnik SF^47^	71	30 (42.25%)	22 (30.99%)	19 (26.76%)
Jaffer S^48^	104	87 (83.65%)	8 (7.69%)	9 (8.65%)
Ahmadiyeh N^13^	69	28 (40.58%)	31 (44.93%)	10 (14.49%)
Sohn YM^49^	39	36 (92.21%)	3 (7.69%)	0 (0%)
Jung SY^50^	160	141 (88.12%)	9 (5.62%)	10 (6.25%)
Youk JH^51^	160	143 (89.38%)	9 (5.63%)	8 (5.0%)
Chang JM^52^	60	46 (76.67%)	12 (20.2%)	2 (3.33%)
Rozentsvavg E^53^	67	54 (80.60%)	8 (11.94%)	5 (7.46%)
Rizzo M^54^	276	197 (71.38%)	42 (15.22%)	37 (13.41%)
Jakate K^55^	162	90 (55.56%)	38 (23.46%)	34 (20.99%)
Lu Q^56^	106	69 (69.09%)	25 (23.58%)	12 (11.32%)
Fu CY^30^	280	174 (62.14%)	72 (25.71%)	34 (12.14%)
Al Hassan T^18^	130	90 (69.23%)	23 (17.69%)	17 (13.08%)
Chang JM^57^	64	55 (85.94%)	7 (10.94%)	2 (1.56%)
Glenn ME^29^	179	114 (63.69%)	43 (24.02%)	22 (12.29)
Nakhlis F^3^	97	42 (43.30%)	41 (42.27%)	14 (14.43%)
Foley NM^58^	238	156 (65.55%)	37 (15.55%)	45 (18.91%)
Boufelli G^59^	85	80 (94.12%)	0 (0%)	5 (5.88%)
Niinikoski L^60^	28	25 (89.29%)	2 (7.14%)	1 (3.57%)
Tran HT^5^	58	43 (74.24%)	10 (17.24%)	5 (8.62%)
Total	2990	2094 (70.03%)	516 (17.26%)	238 (12.71%)

In our research, most women experienced asymptomatic lesions, pain, or discomfort in their breast without nipple discharge or a palpable mass. If the recommended breast imaging indicated ultrasound abnormality or mammographic distortion, the risk assessment of breast malignancy was performed. If necessary, standard treatment for this condition involved surgery to remove the abnormality, especially the papilloma and the affected part of the milk duct. The diagnosed papilloma was removed by excision. The patient's demographics and clinical features were extracted and retrospectively reviewed from the medical records. The clinical presentation of ductal papilloma varied from asymptomatic, nipple discharge, palpable mass, breast pain, or discomfort. Based on our data, 41.30% of intraductal breast papilloma had nipple discharge, 16.22% had a palpable breast mass, and 28.18% were asymptomatic or only had breast discomfort. In addition, intraductal breast papilloma with malignancy had significant correlations with clinical manifestations such as nipple discharge, a palpable breast mass, ultrasound abnormality (BIRADS 4C and 5), mammographic distortion, and microcalcification upon mammography. Clinical manifestations, supersonic findings, and microcalcification upon mammography were significantly associated with the coexisting lesions of malignancy. In our results, coexisting AH associated with nipple discharge, with abnormality by ultrasound, or with microcalcification upon mammography compared with the benign lesions. It is not easy to distinguish the coexisting benign lesion with papilloma from the occulted malignancy or ADH by clinical manifestations. Coexisting AH was also correlated with abnormalities found by ultrasound or microcalcification compared with the benign lesions. It need the professional mammogram and breast ultrasound in clinic evaluated by talented radiologist.

Evidence supporting the excision of papilloma with AH is certainly incontrovertible.^21^, ^22^ The treatment of intraductal papilloma without atypia is controversial and presents the notion, to observe or to excise? Some researchers have advocated that if the radiologic and pathologic results are concordant, regular clinical and radiologic follow‐up are safe for the treatment of intraductal papilloma without atypia,^23^, ^24^, ^25^, ^26^, ^27^, ^28^ whereas others suggest surgical resection for this group of patients to avoid the possibility of breast cancer.^17^, ^29^, ^30^, ^31^ As is known, CNB‐proven intraductal papilloma is removed by CNB, either partly or completely. This approach is the diagnostic challenge in papilloma of the breast. The sensitivity of CNB was based on the complete removal of the representative sampling of the target and thus was partly insufficient. A larger CNB sample can identify breast papillary lesions and can identify patients who do not need surgical resection. With CNB, Shamonki J et al reported that they identified 51 patients with benign papilloma without atypia who subsequently had surgical resection.^32^ A total of 11.7% (6/51) of those who had excision revealed ADH, DCIS, or invasive carcinoma near the papilloma. Among the malignant cases, one excision showed DCIS within the residual papilloma, one excision showed DCIS within the ducts surrounding the papilloma and within an immediately adjacent 0.15‐cm invasive carcinoma, and one excision revealed ADH within the excised papilloma with an adjacent incidental 0.1‐cm tubular carcinoma. These results indicate that excision of a larger tissue sample with CNB can significantly improve the predictive value for benign lesions. Several investigators reported that papillary lesions sampled with larger vacuum‐assisted excision (VAE) have lower upgrade rates and it is suggested that surgical resection may not be necessary in many of these cases.^33^, ^34^, ^35^ VAE provided a method with 9 or 11 needles to obtain more tissue than an automated large‐core biopsy device that usually has a 14‐gauge needle.^36^, ^37^, ^38^ Research from Korea found that the US 14G automated core needle biopsy (ACNB) has a higher false‐negative rate and histological upgrading rate in the diagnosis for papillary breast lesions than US‐guided VAE.^34^ Kim SY et al point out that for asymptomatic benign papilloma, if it has benign or low suspicious ultrasound features or imaging is consistent with pathology, it does not need to be resected immediately and can be followed up.^33^ The researchers included 197 women, of whom 230 cases of asymptomatic benign papilloma were diagnosed by ultrasound‐guided CNB and, if necessary, immunohistochemical staining. A total of 144 women underwent surgery, 86 of whom had a VAE and after the benign VAE results all the patients were followed up for at least 12 months. Eighty‐six women treated with VAE were followed up for an average of 26.3 ± 10.3 months (mean range 12 to 46 months). No sign of malignant tumor was found in the same quadrant. Nayak A points out that conservative treatment may be more appropriate for women diagnosed papilloma by CNB with adequate sampling and precise pathological/radiological correlation, especially those who have received VAE treatment.^23^ When the patient has symptoms or lesions greater than 1.5 cm, the recommended surgical indications include pathological/radiological inconsistencies or ultrasound‐guided CNB sampling without vacuum assistance.

Intraductal papilloma can be single or multiple, located around the breast and can be found occasionally on imaging.^39^, ^40^ A study has shown that for the treatment of benign intraductal solitary papilloma diagnosed by CNB, instead of mandated surgical resection, clinical follow‐up can be selected when the imaging results are consistent and there are no related high‐risk lesions or concurrent malignant tumors in the same quadrant.^24^ This study is important for the research on solitary intraductal papillomas. A question that remains is how to determine solitary or multiple intraductal papillomas, whether by sonography, pathology, or both. Clinically, multiple papilloma is defined as the presence of at least five distinct independent papillomas in a segment of breast tissue, usually around or under the areola.^2^, ^41^ In ultrasound, solitary papilloma can be seen as an independent mass in the dilated mammary duct, a cystic or solid mass with a clear boundary.^41^, ^42^ Yi W et al confirmed single papilloma by ultrasound,^43^ and Zhu Y et al defined it with a histopathologic diagnosis of solitary intraductal papilloma.^44^ Clinically, there are discordant conclusions between sonography and pathology. Our study was limited, owing to the vague definition regarding solitary or multiple intraductal papillomas. We did not distinguish the papilloma by the solitary or multiple lesion criteria.

What are the recommendations for papillomas, or the intermediated risk lesions? The intraductal papilla of the breast represents a series of lesions, the pathological basis of which is the proliferation of intraductal epithelium and myoepithelial cells, covering the fibrous vascular stalk. For the diagnostically challenging intraductal papilloma with limited sampling via CNB, all papillary lesions should be resected for definite diagnosis. The entire lesion may need to be evaluated to exclude occult AH or malignancy. Therefore, the surgical excision is clearly justified. If the clinical evaluation suggests the high probability of papilloma, we recommended the clinical evaluation of its range and coexisted lesions also. If the evaluated range of papilloma with benign lesions is small and limited which can be removed by large VAE, we recommend the use of ultrasound‐guided excision to achieve both diagnosis and treatment of breast intraductal papillary lesions. Large VAE is a suitable choice and a strict clinical follow‐up is essential. The discordance between pathology and radiology leads to the upgrade rate of malignancy as well as unnecessary surgical incision. In the future, it is time for an integration of radiology and pathology. In our research, ultrasound was a very useful tool in the preoperative evaluation of breast papilloma and its coexisting lesions, and mammography was powerful for small nodules or clusters of microcalcifications. For an asymptomatic patient with intracystic hypoechoic lesions as seen by ultrasound, a reexamination 1 month later is suggested to eliminate intracystic deposits. It is obvious that any improvements to resolving the discordance between radiology and pathology and to ensure the timely exchange of clinical features could reduce false‐negative results and unnecessary surgical excision. The improvement of imaging technology can help us diagnose breast lesions more accurately before surgery and afford valuable information. Further studies investigating papilloma are needed.

## 4 COMPETING INTERESTS

The authors declared that they have no competing interests.

## 5 ETHICS STATEMENT

The clinicopathological features of each case were reviewed carefully. Informed consent was exempted due to the retrospective format of this study before 2011. All the patients who were selected in this research signed an informed consent form for the agreement of participation in this research and for the publication of the results. The research was reviewed and approved by The Ethics Committee of China Medical University and The First Affiliated Hospital of China Medical University.

## DISCLOSURE

Only the abstract of our manuscript (A retrospective observational study of intraductal breast papilloma and its coexisting lesions: a real‐world experience) had been submitted to 2018 ASCO meeting (Abstract No: e13558), it is publication‐only abstracts. The whole manuscript had not been submitted to other journals.

## AUTHOR CONTRIBUTIONS

XM and HW wrote the manuscript. XL, CF, and XM collected the data. HW and ZS analyzed the data. FJ and XM supervised the research.
